# Prognostic value of serum lactate kinetics in critically ill patients with cirrhosis and acute-on-chronic liver failure: a multicenter study

**DOI:** 10.18632/aging.102062

**Published:** 2019-07-01

**Authors:** Feng Gao, Xie-lin Huang, Meng-Xing Cai, Miao-tong Lin, Bin-feng Wang, Wei Wu, Zhi-Ming Huang

**Affiliations:** 1 Department of Gastroenterology and Hepatology, The First Affiliated Hospital of Wenzhou Medical University, Wenzhou, China; 2 Department of Gastroenterology Surgery, The Second Affiliated Hospital of Wenzhou Medical University, Wenzhou, China; 3 Department of Cardiovascular Medicine, The Heart Center, The First Affiliated Hospital of Wenzhou Medical University, Wenzhou, China; 4 Department of Emergency Medicine, Intensive Care, The First Affiliated Hospital of Wenzhou Medical University, Wenzhou, China

**Keywords:** lactate, lactate clearance, cirrhosis, acute-on-chronic liver failure, mortality

## Abstract

Lactate clearance (Δ24Lac) was reported to be inversely associated with mortality in critically ill patients. The aim of our study was to assess the value of Δ24Lac for the prognosis of critically ill patients with cirrhosis and acute-on-chronic liver failure (ACLF). We analysed 954 cirrhotic patients with hyperlactatemia admitted to intensive care units (ICUs) in the United States and eastern China. The patients were followed up for at least 1 year. In the unadjusted model, we observed a 15% decrease in hospital mortality with each 10% increase in Δ24Lac. In the fully adjusted model, the relationship between the risk of death and Δ24Lac remained statistically significant (hospital mortality: odds ratio [OR] 0.84, 95% confidence interval [CI]: 0.78- 0.90, p < 0.001; 90-day mortality: hazard ratio [HR] 0.94, 95%CI 0.92- 0.97, p < 0.001; for Δ24Lac per 10% increase). Similar results were found in patients with ACLF. We developed a Δ24Lac-adjusted score (LiFe-Δ24Lac), which performed significantly better in the area under the receiver operating characteristic curves (AUROCs) than the original LiFe score for predicting mortality. Lactate clearance is an independent predictor of death, and the LiFe-Δ24Lac score is a practical tool for stratifying the risk of death.

## INTRODUCTION

Lactate can be measured in critically ill patients to evaluate the severity of disease [1–3]. Patients are considered to have higher lactate levels (hyperlactatemia) at concentrations of more than 2 mmol/L. Hyperlactatemia occurs when lactate production exceeds clearance [4]. Tissue hypoxia and subsequent anaerobic metabolism are considered to be the main mechanisms of hyperlactatemia. Increased lactate production and reduced lactate clearance are common and associated with high mortality in critically ill patients. Studies show that dynamic lactate measures in the intensive care unit (ICU) are better than static lactate measurements for predicting deaths [5–7]. Recently, Masyuk et al. used maximum lactate levels at day 1 and day 2 to calculate lactate clearance (Δ24Lac) and reported that lower Δ24Lac was strongly associated with increased mortality in critically ill patients [8].

Liver cirrhosis is considered an irreversible end result of chronic liver diseases [9]. Hospital mortality of cirrhotic patients admitted to the ICU ranges from 34 to 86% [10]. The combination of decompensated cirrhosis, organ failure(s) and high mortality rate marks the diagnosis of acute-on-chronic liver failure (ACLF). Hyperlactatemia upon admission to the ICU was strongly associated with adverse outcomes in cirrhotic patients [11]. The liver exhibits a higher net lactate clearance than any other organ, accounting for up to 70% of lactate clearance [12]. Lactate kinetics in cirrhotic patients are significantly different from those in patients without hepatic impairment [13]. Fulminant liver dysfunction has been shown to impair lactate clearance [14].

Several scoring systems incorporating lactate levels have been established to evaluate the prognosis of patients with cirrhosis and ACLF [15–18]. The liver injury and failure evaluation (LiFe) score, which is calculated using arterial lactate, serves as a useful tool for predicting the mortality of critically ill patients with cirrhosis and ACLF [17, 18].

A number of studies have demonstrated that lactate is a reliable prognostic marker in the intensive care setting to identify cirrhotic patients at high risk of death. However, few studies focused on the prognostic value of lactate clearance in critically ill patients with cirrhosis and ACLF. Our study aimed to evaluate the prognostic value of lactate clearance in critically ill cirrhotic patients with hyperlactatemia admitted to the ICU. Moreover, we aimed to evaluate whether incorporation of lactate clearance instead of lactate into the LiFe score for patients with cirrhosis and ACLF may help to improve the prognostic performance.

## RESULTS

### Baseline characteristics of the included patients

In the derivation cohort (MIMIC cohort), most patients were male (65.0%) and white (70.9%). Mean age was 58 ± 13 years. Alcoholic liver disease was the main cause of liver cirrhosis, which accounted for 54.5% of cases. The main causes of ICU admission were infections/sepsis, organ failure, and gastrointestinal bleeding. The majority of patients presented with ACLF (69.7%), and ACLF stage 3 was most frequent (31.7%). The median MELD, CLIF-SOFA, and CLIF-C ACLF scores at admission were 25 (19–34), 11 (8–14), and 55 (48–62), respectively. Most patients received vasopressors (60.8%) and mechanical ventilation (77.6%). The median length of ICU stay was 5 (3–10) days. In-hospital mortality was 40.3%, all-cause 28-day mortality was 39.9%, 90-day mortality was 49.4%, and 1-year mortality was 56.2%.

There were two validation cohorts (eICU cohort and WMU cohort) in our study. Patients who came from the eICU cohort had similar baseline characteristics to patients in the MIMIC cohort. In the WMU cohort, all of the participants were Chinese. The main cause of liver cirrhosis was viral hepatitis (59.1%). All of the scores were lower in the WMU cohort, compared to the other two cohorts. Moreover, mortalities in the WMU cohort were also lower than in the other cohorts. There were four patients who underwent liver transplantation within 28 days, and there were nine patients who underwent liver transplantation within 90 days after ICU admission. More information about the baseline characteristics of the patients in the three cohorts is listed in Table 1.

**Table 1:** Patients’ characteristics of the three cohorts.

**Characteristic**	**MIMIC cohort N=429**	**eICU cohort N=303**	**WMU cohort N=222**
**Age, year**	58 ± 13	57 ± 12	59 ± 13
**Sex, male (%)**	65.0%	65.3%	78.4%
**Ethnicity, (%)**			
White	70.9%	74.1%	
Black	10.0%	7.9%	
Asians	1.9%	1.0%	100%
Others	17.2%	17.0%	
**Cause of cirrhosis, (%)**			
Alcoholic	54.5%	56.7%	16.7%
Viral infection	19.8%	18.6%	59.1%
Nonalcoholic steatohepatitis	22.6%	19.4%	21.8%
Biliary	1.4%	2.3%	0.1%
Autoimmune	1.7%	3.0%	2.3%
**Causes of ICU admission, (%)**			
Infection/sepsis	73.4%	42.9%	67.1%
Bleeding	14.9%	9.9%	21.1%
Renal failure	23.3%	10.9%	20.6%
Respiratory failure	47.8%	50.1%	53.2%
Hemodynamic failure	37.5%	44.8%	48.4%
Neurological failure	9.8%	3.6%	3.6%
**ACLF stage, (%)**			
No ACLF	30.3%	26.1%	43.2%
ACLF stage 1	13.5%	13.9%	10.4%
ACLF stage 2	24.5%	25.4%	25.7%
ACLF stage 3	31.7%	34.6%	20.7%
**Lactate (mmol/l), median (IQR)**			
Day 1	4.1 (2.8–6.7)	5.3 (3.5–8.2)	5.6 (3.4–10.2)
Day 2	2.5 (1.8–4.0)	2.9 (2.0–5.7)	3.3 (2.3–5.4)
Day 3–7	2.3 (1.6–3.7)	2.9 (1.9–6.2)	3.4 (2.4–6.0)
Δ24Lac, %	31 (3–56)	30 (0–57)	27 (0–52)
ΔLac_3–7_, %	38 (4–63)	36 (0–65)	25 (0–52)
**Scoring systems, median (IQR)**			
LIFE score	4 (3– 6)	5 (3–6)	3 (2–5)
MELD score	25 (19–34)	21 (14–29)	19 (13–28)
SOFA	12 (9–14)	11 (8–14)	11 (8–13)
CLIF-SOFA	11 (8–14)	9 (7–12)	8 (5–11)
CLIF-C ACLF score	55 (48–62)	58 (51–63)	50 (42–59)
Child-Pugh grade, (%)			
Grade A	7.9%	4.3%	10.4%
Grade B	34.5%	30.0%	36.4%
Grade C	57.6%	65.7%	43.2%
**Therapy, (%)**			
Vasopressor used	60.8%	52.1%	68.4%
Mechanical ventilation	77.6%	41.9%	50.9%
Renal replacement therapy	13.1%	6.9%	22.1%
**Outcome**			
Length ICU stay, day	5 (3–10)	4 (2–7)	6 (4–10)
In-hospital mortality, (%)	40.3%	40.6%	13.5%
All cause 28-day mortality, (%)	39.9%	NA	24.8%
All cause 90-day mortality, (%)	49.4%	NA	30.1%
All cause 1-year mortality, (%)	56.2%	NA	35.6%

Note: Δ24Lac: lactate clearance; ACLF: acute-on-chronic liver failure; CLIF-SOFA: chronic liver failure (CLIF)-SOFA; ICU: intensive care unit; LiFe: liver injury and failure evaluation; MELD: model for end-stage liver disease; SOFA: sequential organ failure assessment.

**Figure 1:**
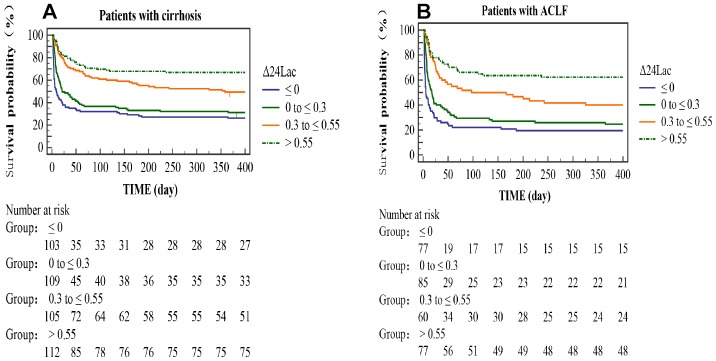
**Kaplan-Meier curves stratified by Δ24Lac quartiles.** The curves showed different the cumulative survival rates of patients with different Δ24Lac levels. (**A**): cirrhotic patients. (**B**): ACLF patients.

Patients were stratified by Δ24Lac quartiles in the derivation cohort. As presented in Table 2, Δ24Lac levels were positively associated with MAP levels, body temperature levels, 24-hour urine output, and mechanical ventilation support and were inversely associated with bilirubin levels, vasopressor use, and mortality. Figure 1 shows Kaplan-Meier curves illustrating the cumulative survival rates stratified by Δ24Lac quartiles.

**Table 2:** Baseline characteristics of patients by lactate clearance quartiles (derivation cohort).

**Characteristics**	**Δ24Lac ≤ 0 N= 103**	**Δ24Lac 0 to ≤ 0.3 N= 109**	**Δ24Lac 0.3 to ≤ 0.55 N= 105**	**Δ24Lac > 0.55 N= 112**	P value*
**Age (year)**	56.7 ± 10.9	57.2 ± 11.4	59.1 ± 13.2	58.9 ± 14.7	0.69
**Sex, male (%)**	59 (57.3%)	73 (67.0%)	71 (67.6%)	76 (67.9%)	0.31
**Vital signs, median (IQR)**					
Mean arterial pressure (mmHg)	70 (65–77)	72 (66–77)	76 (69–83)	78 (71–90)	<0.01
Body temperature (C°)	36.5 (36.1–37.1)	36.6 (36.2–37.0)	36.6 (36.2–37.1)	37.0 (36.5–37.3)	<0.01
Heart rate (bpm)	90 (77–108)	93 (83–105)	90 (81–104)	94 (82–106)	0.54
24-hour urine output (ml)	710 (314–1265)	740 (224–1364)	1055 (547–1607)	1194 (657–2163)	<0.01
**Laboratory parameters, median (IQR)**					
Albumin (mg/dl)	2.65 (2.23–2.98)	2.70 (2.18–3.02)	2.50 (2.00–3.00)	2.70 (2.35–3.10)	0.22
Bilirubin (mg/dl)	5.70 (2.58–12.20)	5.60 (2.60–11.40)	3.40 (2.10– 5.60)	4.70 (2.70– 8.25)	<0.01
Creatinine (mg/dl)	1.70 (1.00–3.15)	2.10 (1.20–3.40)	1.50 (1.10–2.60)	1.40 (1.00–2.20)	0.04
INR	2.15 (1.80–2.70)	2.20 (1.80–3.10)	1.90 (1.60–2.50)	2.10 (1.70–2.60)	0.05
Hemoglobin, mg/dl	8.7 (7.6–10.1)	8.8 (7.5–10.3)	8.7 (7.6–10.0)	8.5 (7.5– 9.4)	0.35
Platelet, 10^9^/L	75 (53–143)	68 (50–103)	76 (51–113)	68 (51–102)	0.07
WBC, 10^9^/L	12.6 (7.2–21.3)	13.5 (9.0–19.0)	13.1 (8.5–21.7)	12.7 (9.6–17.8)	0.72
Lactate (mmol/l)					
Day 1	3.10 (2.40–6.50)	3.70 (2.50–5.90)	3.70 (2.70–4.80)	5.95 (4.57–8.60)	<0.01
Day 2	4.10 (3.25–8.55)	3.00 (2.20–4.90)	2.00 (1.60–2.90)	1.70 (1.30–2.23)	<0.01
**Characteristics of cirrhosis (%)**					
Infection	87 (84.5%)	88 (80.7%)	71 (67.6%)	69 (61.6%)	<0.01
Hepatic encephalopathy	30 (29.1%)	30 (27.5%)	23 (21.9%)	26 (23.2%)	0.58
Variceal bleeding	35 (34.0%)	30 (27.5%)	29 (27.6%)	34 (30.4%)	0.71
Ascites	27 (26.2%)	35 (32.1%)	31 (29.5%)	32 (28.6%)	0.82
Hepatorenal syndrome	20 (19.4%)	26 (23.9%)	17 (16.2%)	22 (19.6%)	0.57
**Therapy (%)**					
Vasopressor used	75 (72.8%)	68 (62.4%)	65 (61.9%)	53 (47.3%)	<0.01
Mechanical ventilation	73 (70.9%)	80 (73.4%)	83 (79.0%)	97 (86.6%)	0.03
Renal replacement therapy	16 (15.5%)	15 (13.8%)	11 (10.5%)	14 (12.5%)	0.74
**Scoring systems, median (IQR)**					
LIFE score	5 (2–5)	5 (3–5)	3 (2–5)	5 (3–6)	<0.01
MELD score	26 (20–37)	28 (21–39)	22 (18–29)	25 (19–32)	<0.01
SOFA score	12 (9–16)	12 (9–15)	11 (8–13)	11 (8–14)	0.02
CLIF-SOFA score	12 (8–15)	12 (10–14)	10 (8–12)	11 (8–13)	<0.01
CLIF-C ACLF score	57.0 (48.5–63.0)	55.8 (49.5–63.8)	53.0 (47.8–58.8)	53.2 (46.5–61.3)	0.04
Child-Pugh (%)					0.57
Grade A	4 (3.9%)	9 (8.3%)	11 (10.5%)	10 (8.9%)	
Grade B	36 (35.0%)	36 (33.0%)	40 (38.1%)	36 (32.1%)	
Grade C	63 (61.2%)	64 (58.7%)	54 (51.4%)	66 (58.9%)	
**ACLF stage (%)**					0.01
No ACLF	26 (25.2%)	24 (22.0%)	45 (42.9%)	35 (31.2%)	
ACLF stage 1	12 (11.7%)	15 (13.8%)	15 (14.3%)	16 (14.3%)	
ACLF stage 2	29 (28.2%)	22 (20.2%)	24 (22.9%)	30 (26.8%)	
ACLF stage 3	36 (35.0%)	48 (44.0%)	21 (20.0%)	31 (27.7%)	
**Outcome**					
Length ICU stay (day)	4.8 (2.7– 9.2)	6.0 (2.9–11.6)	5.2 (3.3–10.8)	5.4 (3.3–10.6)	0.49
In-hospital mortality (%)	67 (65.0%)	56 (51.4%)	29 (27.6%)	21 (18.8%)	<0.01
All cause 28-day mortality (%)	67 (63.81%)	55 (51.40%)	28 (26.67%)	21 (18.75%)	<0.01
All cause 90-day mortality (%)	71 (67.62%)	68 (63.55%)	40 (38.10%)	33 (29.46%)	<0.01
All cause 1-year mortality (%)	76 (73.8%)	75 (68.8%)	53 (50.5%)	37 (33.0%)	<0.01

Note: INR: international normalized ratio; IQR: interquartile range; WBC: white blood cell count.

* Kruskal-Wallis test was used for continuous variables, and Chi-squared test was used for categorical variables.

### Independent effect of lactate clearance on mortality

In the derivation cohort, we saw significant inverse associations between increasing Δ24Lac and in-hospital mortality. The unadjusted OR for every ten percent increase in Δ24Lac was 0.85 (95% CI 0.81–0.90; P <0.01). After multivariable adjustment, the OR for every ten percent increase in Δ24Lac was 0.84 (95% CI 0.78–0.90; P <0.01). The OR for in-hospital death (vs. Δ24Lac ≤ 0) was 0.40 (95% CI 0.17–0.93) for Δ24Lac 0 to ≤ 0.3; 0.17 (95% CI, 0.07–0.41) for Δ24Lac 0.3 to ≤ 0.55; and 0.03 (95% CI, 0.01–0.10) for Δ24Lac > 0.55 (P for trend <0.001). Δ24Lac was also independently associated with in-hospital mortality in patients with ACLF (Table 3). Further, through Cox regression, we demonstrated that decreased Δ24Lac was significantly associated with increased 28-day and 90-day mortality rates (Table 4 and Supplementary Table 2). In the competing risk analysis (WMU cohort), we also found that Δ24Lac was independently correlated with increased mortality rates (Supplementary Table 3). Each 10% increase in Δ24Lac led to 10% decrease in 90-day mortality.

**Table 3:** Multivariate logistic regression for effect of delta-lactate on in-hospital mortality (derivation cohort).

	**Crude**		**Adjusted model I**		**Adjusted model II**	
	**OR (95% CI)**	**P value**	**OR (95% CI)**	**P value**	**OR (95% CI)**	**P value**
**Cirrhosis**						
Δ24Lac, per 10%	0.85 (0.81, 0.90)	<0.001	0.81 (0.76, 0.86)	<0.001	0.84 (0.78, 0.90)	<0.001
Categories						
Δ24Lac Q1	Ref	Ref	Ref	Ref	Ref	Ref
Δ24Lac Q2	0.57 (0.33, 0.99)	0.045	0.46 (0.25, 0.87)	0.017	0.40 (0.17, 0.93)	0.033
Δ24Lac Q3	0.21 (0.11, 0.37)	<0.001	0.18 (0.09, 0.35)	<0.001	0.17 (0.07, 0.41)	<0.001
Δ24Lac Q4	0.12 (0.07, 0.23)	<0.001	0.04 (0.02, 0.10)	<0.001	0.03 (0.01, 0.10)	<0.001
P for trend		<0.001		<0.001		<0.001
**ACLF**						
Δ24Lac, per 10%	0.84 (0.79, 0.90)	<0.001	0.78 (0.72, 0.84)	<0.001	0.82 (0.75, 0.90)	<0.001
Categories						
Δ24Lac Q1	Ref	Ref	Ref	Ref	Ref	Ref
Δ24Lac Q2	0.48 (0.25, 0.93)	0.030	0.38 (0.18, 0.83)	0.015	0.31 (0.11, 0.91)	0.033
Δ24Lac Q3	0.20 (0.10, 0.42)	<0.001	0.14 (0.06, 0.34)	<0.001	0.12 (0.04, 0.37)	<0.001
Δ24Lac Q4	0.11 (0.06, 0.24)	<0.001	0.03 (0.01, 0.09)	<0.001	0.03 (0.01, 0.11)	<0.001
P for trend		<0.001		<0.001		<0.001

Model I adjusted for age, sex, ethnicity, LiFe score.

Model II adjusted for age, sex, ethnicity, MAP, temperature, 24-hour urine output, albumin, bilirubin, creatinine, INR, WBC, lactate, infection, hepatic encephalopathy, variceal bleeding, ascites, hepatorenal syndrome, vasopressor used, mechanical ventilation, RRT.

**Table 4:** Multivariate Cox regression for effect of lactate-clearance on 90-day mortality (derivation cohort).

	**Crude**		**Adjusted model I**		**Adjusted model II**	
	**HR (95% CI)**	**P value**	**HR (95% CI)**	**P value**	**HR (95% CI)**	**P value**
**Cirrhosis**						
Δ24Lac per 10%	0.94 (0.92, 0.95)	<0.001	0.93 (0.91, 0.94)	<0.001	0.94 (0.92, 0.97)	<0.001
Categories						
Δ24Lac Q1	Ref	Ref	Ref	Ref	Ref	Ref
Δ24Lac Q2	0.73 (0.52, 1.02)	0.066	0.67 (0.48, 0.95)	0.029	0.62 (0.41, 0.95)	0.026
Δ24Lac Q3	0.34 (0.23, 0.50)	<0.001	0.29 (0.19, 0.43)	<0.001	0.32 (0.20, 0.52)	<0.001
Δ24Lac Q4	0.25 (0.16, 0.38)	<0.001	0.14 (0.09, 0.22)	<0.001	0.25 (0.15, 0.43)	<0.001
P for trend		<0.001		<0.001		<0.001
**ACLF**						
Δ24Lac per 10%	0.94 (0.93, 0.96)	<0.001	0.93 (0.91, 0.94)	<0.001	0.93 (0.91, 0.96)	<0.001
Categories						
Δ24Lac Q1	Ref	Ref	Ref	Ref	Ref	Ref
Δ24Lac Q2	0.74 (0.51, 1.07)	0.111	0.70 (0.47, 1.03)	0.072	0.58 (0.36, 0.94)	0.026
Δ24Lac Q3	0.37 (0.24, 0.56)	<0.001	0.29 (0.19, 0.45)	<0.001	0.30 (0.17, 0.51)	<0.001
Δ24Lac Q4	0.22 (0.14, 0.35)	<0.001	0.12 (0.07, 0.19)	<0.001	0.19 (0.10, 0.37)	<0.001
P for trend		<0.001		<0.001		<0.001

Model I adjusted for age, sex, LiFe score.

Model II adjusted for age, sex, mean arterial pressure, temperature, 24-hour urine output, albumin, bilirubin, creatinine, INR, vasopressor used, mechanical ventilation, renal replacement therapy.

We repeated the lactate measurements at day 3-7 and calculated the lactate clearance (ΔLac_3-7_). We demonstrated that ΔLac_3-7_ was also an independent and significant predictor for poor prognosis in critically ill patients with cirrhosis and ACLF (Supplementary Table 4).

### Development and validation of lactate clearance -adjusted LiFe (LiFe-Δ24Lac)

Lactate clearance was identified as a significant predictor of mortality, independent of the LiFe score (Tables 3 and 4). We hypothesized that the addition of lactate clearance instead of lactate to the LiFe score may improve the performance of the score for predicting mortality. The LiFe-Δ24Lac score was calculated by adding points for each of the following risk factors: total bilirubin 0–1.9, ≥2.0–3.9, ≥4.0–5.9, ≥6.0 mg/dL; INR 0–1.9, ≥2.0–3.9, ≥4.0–5.9, ≥6.0; and lactate clearance >0.55, >0.30–0.55, >0–0.30, ≤ 0.

In patients with cirrhosis, AUROCs for LiFe-Δ24Lac in the prediction of hospital, 28-day, 90-day mortality, and 1-year mortality (0.75, 0.74, 0.72 and 0.69) were significantly higher than those obtained for original LiFe score (0.69, 0.67, 0.66 and 0.61, all p < 0.01]). In predicting hospital and 1-year mortality, the addition of Δ24Lac to the LiFe score increased net reclassification improvements (NRIs) by 12.9% (p<0.01) and 16.4% (p<0.01), respectively, and increased integrated discrimination improvements (IDIs) by 8.1% (p=0.03) and 6.6% (p=0.04), respectively.

In patients with ACLF, the LiFe-Δ24Lac score also improved the prediction of mortality, as shown by the significant increase in AUROCs (in-hospital mortality: 0.75; 28-day: 0.74; 90-day: 0.70; 1-year: 0.69) compared with that of the LiFe score (in-hospital mortality: 0.68; 28-day: 0.66; 90-day: 0.63; 1-year: 0.60, all p<0.01). NRIs and IDIs also improved significantly after adding Δ24Lac (in-hospital mortality: NRI 16.4% [p<0.01]; IDI 8.8%, [p=0.04]; 1-year mortality: NRI: 20.2%, [p<0.01]; IDI 7.8%, [p=0.05]).

**Figure 2:**
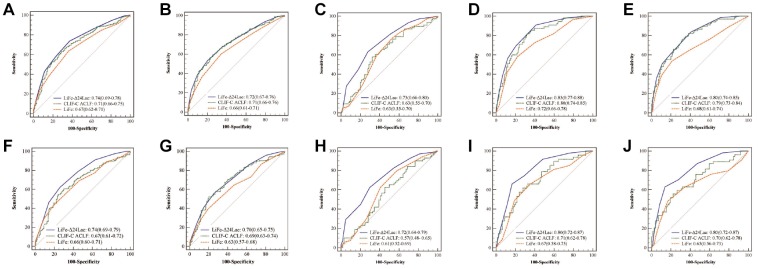
**AUROCs for LiFe-Δ24Lac, CLIF-C ACLF and LiFe scores in prediction of mortality in patients with cirrhosis and ACLF in the derivation and validation cohort.** Cirrhosis patients, MIMIC cohort: 28-day mortality (**A**); 90-day mortality (**B**); eICU cohort: hospital mortality (**C**); WMU cohort: 28-day mortality (**D**); 90-day mortality (**E**). ACLF patients: MIMIC cohort: 28-day mortality (**F**); 90-day mortality (**G**); eICU cohort: hospital mortality (**H**). WMU cohort: 28-day mortality (**I**); 90-day mortality (**J**).

We also compared LiFe-Δ24Lac score with CLIF-C-ACLF score. LiFe-Δ24Lac score performed with a higher discrimination than CLIF-C ACLF score, especially for predicting short-term mortality (hospital and 28-day mortality, p<0.05). The prognostic performance of these scoring systems in the derivation and validation cohort are illustrated in Figure 2. The calibration plots showed good agreement between the LiFe-Δ24Lac score prediction and actual observation in both the primary and validation cohorts (Supplementary Figure 1).

### Outcome events

We re-grouped the LiFe-Δ24Lac scores into three categories (relatively low-risk: 0–3; intermediate-risk: 4–6; and high-risk: 7–9) using X-tile software. In the derivation cohort, the in-hospital, 28-day and 90-day mortality rates were respectively 21.7% (45/207), 21.7% (45/207) and 33.8%(70/207) for patients with low risk; 51.5% (86/167), 50.9%(85/167) and 58.7% (98/167) for patients with middle risk; and 76.4% (42/55), 74.5%(41/55) and 80.0% (44/55) for patients with high risk. More detailed information about outcome events is presented in Table 5.

**Table 5:** The mortality information in patients with cirrhosis and ACLF, according to LiFe-Δ24Lac stratification.

	**In-hospital mortality**			**28-day mortality**		**90-day mortality**	
	**MIMIC cohort**	**eICU cohort**	**WMU cohort**	**MIMIC cohort**	**WMU cohort**	**MIMIC cohort**	**WMU cohort**
**Cirrhosis**							
Low risk	45/207 (21.7%)	30/134 (22.4%)	12/150 (8.0%)	45/207 (21.7%)	17/150 (11.3%)	70/207 (33.8%)	26/150 (17.3%)
Middle risk	86/167 (51.5%)	63/129 (48.8%)	12/61 (19.7%)	85/167 (50.9%)	30/61 (49.2%)	98/167 (58.7%)	32/61 (52.5%)
High risk	42/55 (76.4%)	30/40 (75.0%)	6/11 (55.0%)	41/55 (74.5%)	8/11 (72.7%)	44/55 (80.0%)	9/11 (81.8%)
P for trend	<0.001						
**ACLF**							
Low risk	32/116 (27.6%)	22/85 (25.9%)	9/68 (13.2%)	31/116 (26.7%)	12/68 (17.6%)	49/116 (42.2%)	16/68 (23.5%)
Middle risk	76/133 (57.1%)	53/104 (51.0%)	11/47 (23.4%)	76/133 (57.1%)	27/47 (57.4%)	85/133 (63.9%)	29/47 (61.7%)
High risk	39/50 (78.0%)	29/35 (82.9%)	6/11 (55.0%)	38/50 (76.0%)	8/11 (72.7%)	41/50 (82.0%)	9/11 (81.8%)
P for trend	<0.001						

## DISCUSSION

In our study cohort of critically ill patients with cirrhosis and ACLF, we demonstrated that lactate clearance was independently associated with mortality rates after correction for other confounders. In addition, we found that incorporation of lactate clearance instead of lactate into the LiFe score improves the performance of the score for predicting outcome.

Despite aggressive medical interventions, critically ill patients with cirrhosis and ACLF have poor outcomes. Consistent with the findings from our study, the mortality rates of cirrhotic patients with hyperlactatemia admitted to the ICU ranged from 13 to 40% during hospital and ranged from 35 to 56% after 1-year follow-up. Most patients had infection, sepsis, and even multi-organ failures at ICU admission. A recent multicenter study revealed that ICU and hospital mortality rates in critically ill patients with ACLF were 39.2% and 54.6%, respectively [15]. The hospital and 1-year mortality of patients with ACLF in our study were 49.1% and 63.9%. Therefore, quick and accurate assessment of the severity of disease, which can help with timely initiation of organ support to improve prognosis, is urgently needed.

In clinical practice, blood lactate level is a useful marker for predicting the outcomes of critically ill patients, such as patients with sepsis and shock [19]. In different studies, optimal cutoffs of single static arterial lactate measurements vary considerably. In this context, fluctuations of blood lactate levels have attracted the interest of clinicians and researchers in recent years.

Previous studies demonstrated that early changes in lactate levels could be a practical tool for risk assessment [8, 20–22]. They also found that higher lactate clearance is associated with lower mortality rates. A large multicenter randomized controlled trial showed that lactate clearance could guide therapeutic measures in septic patients [23]. However, few studies focused on the prognostic value of lactate clearance in critically ill patients with cirrhosis and ACLF.

Recently, Drolz and colleagues conducted a retrospective study that included 816 critically ill patients with cirrhosis from 3 university hospitals in Europe [16]. They reported for the first time that lactate and lactate clearance were independent predictors of outcome in critically ill patients with cirrhosis and ACLF. Overall, our finding of decreased risk for death with increased lactate clearance is consistent with their results. In our study, risk reductions for hospital mortality were 16% (95%CI 10%–22%) in patients with cirrhosis and 18% (95%CI 10%–25%) in patients with ACLF for each 10% increase in Δ24Lac. In predicting 90-day all-cause mortality, risk reductions were 6% (95%CI 3%–8%) in patients with cirrhosis and 7% (95%CI 4%–9%) in patients with ACLF for each 10% increase in Δ24Lac.

There are, however, several differences between the two studies. First, the patients in our study came from the USA and eastern China. Patients from different regions may have had different baseline characteristics. Second, we calculated Δ24Lac by reference to the method of Masyuk et al [8]. There was no prespecified time-based protocol for lactate measurements in our study. Focusing on maximum lactate concentrations may better reflect real-world lactate measurements. Third, Drolz et al. incorporated lactate into the CLIF-C ACLF score to improve the performance of the score [16], but we added lactate clearance instead of lactate to the LiFe score.

ACLF is a dynamic syndrome. Gustot et al. found that evaluating ACLF patients at 3–7 days after ICU admission provided a tool for predicting the prognosis [24]. Therefore, we repeated the lactate measurements at day 3–7 and calculated the lactate clearance to assure our prediction was right. Through multivariate regression, we demonstrated that higher lactate clearance in patients at day 3–7 was independently associated with lower mortality rates.

We evaluated for the first time the potential contribution of lactate clearance to current scoring systems in a large multinational cohort of critically ill patients with cirrhosis and ACLF. We developed and externally validated a new score containing lactate clearance, named LiFe-Δ24Lac, for critically ill patients with cirrhosis and ACLF. The laboratory-based LiFe-Δ24Lac score is simple and objective and can be calculated quickly at the bedside. The superiority of the new score for predicting mortality rates was supported by a number of tests, such as the AUROC, NRI, IDI, and calibration curves. To improve its feasibility, we divided the score into three levels of risk (relatively low risk, intermediate risk, and high risk). The patients stratified into different groups had significantly different prognoses.

Our study has several limitations. First, we excluded patients who stayed in the ICU for no more than 48 hours and patients without hyperlactatemia. Our findings only can be generalized to patients with hyperlactatemia who stayed in the ICU for more than 48 hours. Second, this is a retrospective study. Limited by its retrospective nature, 289 patients were excluded because of the absence of repeated lactate measurements, which may have increased the selection bias. However, we demonstrated that baseline characteristics of these patients were comparable to those with repeated lactate measurements (Supplementary Table 1). We also externally validated the results in the other two cohorts. These lost cases might have a limited effect on the evaluation of the independent effect of Δ24Lac on the prognosis of cirrhosis. Second, the hospital mortality of the patients in the WMU cohort was significantly lower than in the other two cohorts. This phenomenon could be explained by the fact that most Chinese critically ill patients wish to die at home based on Chinese cultural perspectives. A study conducted in China revealed that 26–44% of adult ICU patients were transported home to die [25]. Third, there was no information about liver transplantation within the 1-year follow-up in the MIMIC database, so we could not conduct competing risk model in this cohort. However, we utilized a competing risk approach in the WMU cohort considering liver transplantation as a competing risk.

In conclusion, lactate clearance is a good and independent predictor of death in critically ill patients with cirrhosis and ACLF. Incorporation of lactate clearance into the current scoring system improved the performance of the score for predicting outcome. LiFe-Δ24Lac is a quick and easy risk stratification score and may help with the timely identification of patients at high-risk of death. More large-scale prospective multicenter studies are needed to evaluate and verify the applicability of this approach.

## METHODS

### Data source

We extracted the patients’ dataset from the Medical Information Mart for Intensive Care (MIMIC)-III and the eICU Collaborative Research Database (eICU) [26, 27]. The MIMIC-III is a database consisting of over 40000 ICU patients who stayed in intensive care units of the Beth Israel Deaconess Medical Center between 2001 and 2012. The eICU database comprises 200,859 ICU patients admitted to 208 hospitals located throughout the United States between 2014 and 2015. Medical history, clinical information, vital signs, and laboratory parameters were documented in these databases. We obtained access to these databases after completing the National Institute of Health’s training course ‘Protecting Human Research Participants’ (certificate number: 25557915).

### Study design

We conducted an international multicenter retrospective cohort study. Adult cirrhotic patients with hyperlactatemia on admission were eligible for inclusion in this study. We excluded patients who stayed in the ICU for no more than 48 hours. The primary endpoint of the study was hospital mortality, and the secondary endpoint was determined considering the all-cause 28-day, 90-day and 1-year mortality rates. The diagnosis of cirrhosis was based on histopathology, ultrasonography or computed tomography findings, clinical evidence of liver dysfunction or portal hypertension. We diagnosed and graded ACLF according to the criteria established by the EASL–CLIF consortium [28]. Hyperlactatemia was defined by arterial lactate levels above 2.0 mmol/L.

**Figure 3:**
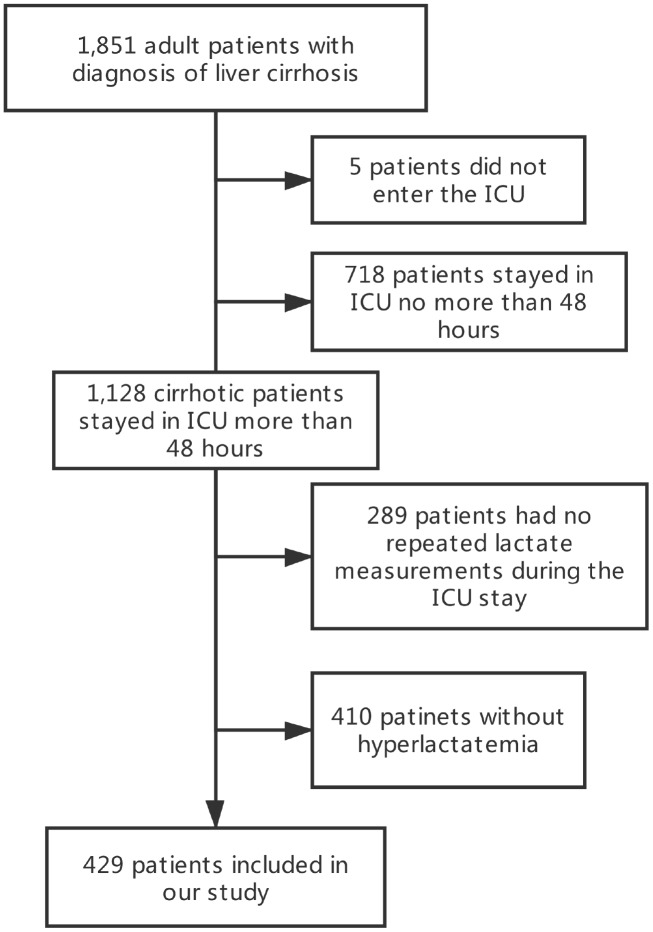
**A flow diagram of study participants (derivation cohort).**

In the derivation cohort, we included 429 cirrhotic patients (7,189 measurements) with hyperlactatemia from the MIMIC database. The flow chart is presented in Figure 3. Additionally, we analysed a validation cohort of 303 patients collected from the eICU database. Another validation cohort (WMU cohort) of 222 cirrhotic patients collected from ICUs of the First Affiliated Hospital of Wenzhou Medical University (Zhejiang, China) between January 2012 and January 2018 was also analysed.

The MIMIC and eICU cohorts’ mortality information came from the social security database *in* *the* *United* *States*. The mortality information of the WMU cohort was collected by review of medical records or by contacting the patients. The ethics committee of the First Affiliated Hospital of Wenzhou Medical University approved this study. All study procedures followed the principles of the Declaration of Helsinki. No informed consent was required because all the data were anonymized.

### Lactate-clearance and scoring systems calculation

The main focus of the study was arterial lactate kinetics over the first 48 hours of admission. To obtain Δ24Lac, we recorded the maximum lactate levels of day 1 and day 2. We calculated Δ24Lac based on the formula: Δ24Lac (%) = (Lactate _day1 max_ – Lactate _day2 max_)/ Lactate _day1 max_[8]. Time-based protocols of lactate concentration determination were not prespecified. This reflected the real-world scenario regarding the timing and frequency of lactate measurements. Although there was no time-based protocol regarding lactate measurements, the lactate values were measured on a relatively regular basis in enrolled patients (at least one time during each 6-h shift on admission day, and at least one time per day during the ICU stay).

We also repeated the measurement on day 3–7 to assure our prediction was right. We calculated the lactate clearance at day 3–7 according to the formula:

$$\Delta Lac_{3-7}\left(\%\right)=\left(Lac_{day1max}–Lac_{day3-7max}\right)/Lac_{day1max}.$$

The Child-Pugh, model of end-stage liver disease (MELD), sequential organ failure assessment (SOFA), chronic liver failure–SOFA (CLIF-SOFA), and CLIF-C ACLFs scores were calculated according to the published formulae [28–32]. The LiFe score was calculated by adding points for each of the following risk factors: total bilirubin 0–1.9, ≥2.0–3.9, ≥4.0–5.9, ≥6.0 mg/dL; INR 0–1.9, ≥2.0–3.9, ≥4.0–5.9, ≥6.0; arterial lactate 0–1.9, ≥2.0–3.9, ≥4.0–5.9, ≥6.0 mg/dL[17].

### Statistical analysis

We categorized lactate clearance as ≤ 0, >0–0.3, >0.3–0.55, and >0.55. Continuous variables were expressed as median with interquartile range (IQR) and compared by Kruskal-Wallis test. Categorical variables were expressed as percentages (%) and compared by Chi-Square test. To maximize data availability, we used multiple imputations, based on 5 replications and a chained equation approach method in the R MI procedure, to account for missing albumin, INR, bilirubin, creatine, and urine output date. We also performed sensitivity analyses using a complete-case analysis. Univariate and multivariate logistic regressions were used to identify the association between lactate clearance and hospital mortality. Odds ratios (OR) were reported with a 95% confidence interval (CI). The association between 28-day and 90-day mortality and lactate clearance was analysed by univariable and multivariable Cox proportional hazards models, and hazard ratios (HR) were calculated. We performed tests for linear trend by entering the median value of the variable. We conducted a multivariate competing risk regression (Fine-Gray model) in the WMU cohort to assess the effect of lactate clearance on mortality. Liver transplantation was taken into account as a competing risk.

We plotted cumulative survival curves by the Kaplan-Meier method and compared the survival curves using the log-rank test. The performances of the scoring systems were assessed by calculation of the area under the receiver- operating characteristic curve (AUROC) and assessed using the DeLong test [33]. Furthermore, we used the net reclassification index (NRI) and integrated discrimination improvement (IDI) to evaluate the additive predictive value of lactate clearance instead of lactate over the LiFe score in assessing the improvement of prognostic value [34]. A calibration curve was used to compare the predicted probability of survival versus actual, using 500 bootstraps resamples to reduce overfit bias. All of the tests were two sided, and a p value of <0.05 was considered statistically significant. The statistical packages R (version 3.4.3, The R Foundation; http://www.r-project.org) and MedCalc (version 15.2.2 Ostend, Belgium) were used for statistical analyses.

The LiFe-Δ24Lac scores were re-grouped into three categories (relatively low-risk, intermediate-risk and high-risk) using X-tile software (Version 3.6.1, Yale University, USA) to calculate the optimal cut-off values[35].

## Supplementary Material

Supplementary Figure

Supplementary Tables
