# Supplementary material for: Prognostic value of serum lactate kinetics in critically ill patients with cirrhosis and acute-on-chronic liver failure: a multicenter study
Source: Aging (Albany NY). 2019 Jul 1;11(13):4446–62. doi: 10.18632/aging.102062 (PMC6660055; doi:10.18632/aging.102062)
Supplement: Supplementary Figure [file aging-11-102062-s001.pdf]

SUPPLEMENTARY FIGURE

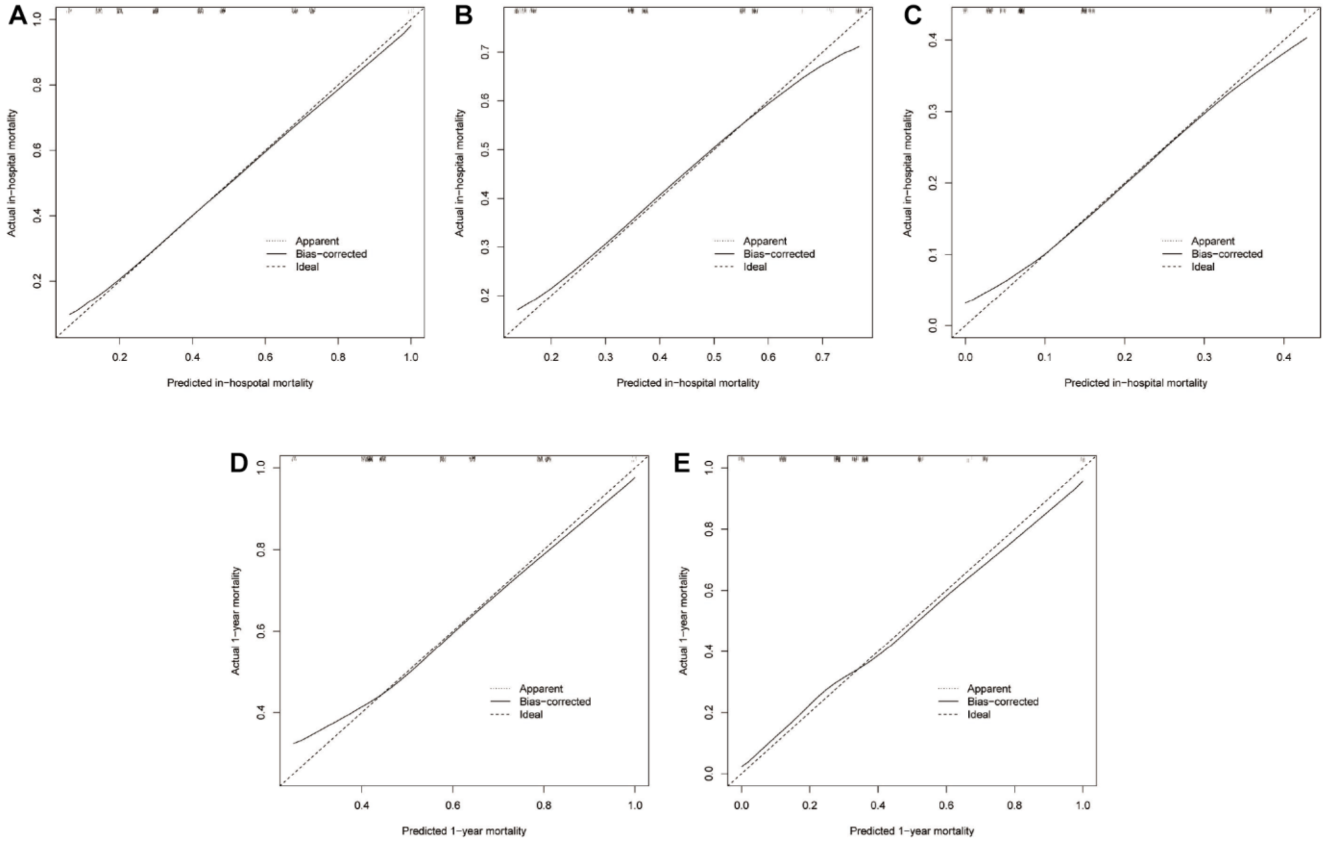

Supplementary Figure 1. Calibration curves of LiFe-Δ24Lac. The y-axis represents the actual mortality rate. The x-axis represents the predicted mortality rate. The shallow line represents a perfect prediction by an ideal model. Predicting in-hospital mortality in patients with cirrhosis: MIMIC cohort (**A**); eICU cohort (**B**); WMU cohort (**C**). Predicting 1-year mortality in patients with cirrhosis: MIMIC cohort (**D**); WMU cohort (**E**).
