# Supplementary material for: Prognostic value of serum lactate kinetics in critically ill patients with cirrhosis and acute-on-chronic liver failure: a multicenter study
Source: Aging (Albany NY). 2019 Jul 1;11(13):4446–62. doi: 10.18632/aging.102062 (PMC6660055; doi:10.18632/aging.102062)
Supplement: Supplementary Tables [file aging-11-102062-s002.pdf]

SUPPLEMENTARY TABLES

Supplementary Table 1. The primary outcome of patients with and without repeated lactate measurements.

Characteristic	Repeated lactate available N=839	Repeated lactate unavailable N=289	P-value
**Age, year**	60 ± 15	60 ± 17	0.91
**Sex, male (%)**	67.7%	65.4%	0.49
**Ethnicity, (%)**			
White	66.0%	66.1%	0.18
Black	10.2%	6.2%	
Asians	3.2%	2.4%	
Others	20.6%	25.3%	
**Cause of cirrhosis, (%)**			
Alcoholic	46.3%	52.9%	0.12
Viral infection	16.3%	13.5%	
Nonalcoholic steatohepatitis	33.3%	32.2%	
Biliary	3.1%	1.4%	
Autoimmune	1.0%	0%	
**Causes of ICU admission, (%)**			
Infection/sepsis	53.9%	59.2%	0.16
Bleeding	10.5%	11.9%	0.55
Renal failure	13.3%	14.5%	0.63
Respiratory failure	29.3%	28.0%	0.57
Hemodynamic failure	17.0%	19.9%	0.13
Neurological failure	3.5%	4.4%	0.50
**ACLF stage, (%)**			
No ACLF	50.1%	48.4%	0.84
ACLF stage 1	16.3%	17.9%	
ACLF stage 2	17.5%	18.9%	
ACLF stage 3	15.1%	14.8%	
**Scoring systems, median (IQR)**			
MELD score	18 (13–26)	19 (14–26)	0.79
SOFA	8 (6–10)	9 (6–11)	0.49
CLIF-SOFA	8 (5–10)	8 (6–10)	0.68
CLIF-C ACLF score	45 (39–52)	46 (38–54)	0.82
Child-Pugh grade, (%)			
Grade A	23.2%	22.9%	0.84
Grade B	40.4%	41.5%	
Grade C	36.4%	35.6%	
**Therapy, (%)**			
Vasopressor used	31.8%	35.6%	0.06
Mechanical ventilation	48.3%	42.6%	0.08
Renal replacement therapy	8.6%	9.4%	0.64
**Outcome**			
In-hospital mortality, (%)	25.6%	27.3%	0.77
All cause 28-day mortality, (%)	30.5%	32.6%	0.52
All cause 90-day mortality, (%)	39.6%	41.5%	0.60
All cause 1-year mortality, (%)	49.5%	51.9%	0.51

Note: Δ24Lac: lactate clearance; ACLF: acute-on-chronic liver failure; CLIF-SOFA: chronic liver failure (CLIF)-SOFA; ICU: intensive care unit; MELD: model for end-stage liver disease; SOFA: sequential organ failure assessment.

Supplementary Table 2. Multivariate Cox regression for effect of lactate-clearance on 28-day mortality (derivation cohort).

	Crude		Adjusted Model I		Adjusted Model II	
	**HR (95% CI)**	**P value**	**HR (95% CI)**	**P value**	**HR (95% CI)**	**P value**
**Cirrhosis**						
Δ24Lac per 10%	0.93 (0.92, 0.95)	<0.001	0.92 (0.91, 0.94)	<0.001	0.94 (0.91, 0.96)	<0.001
Categories						
Δ24Lac Q1	Ref	Ref	Ref	Ref	Ref	Ref
Δ24Lac Q2	0.62 (0.43, 0.89)	0.009	0.55 (0.38, 0.80)	0.002	0.51 (0.32, 0.80)	0.003
Δ24Lac Q3	0.26 (0.17, 0.41)	<0.001	0.23 (0.15, 0.36)	<0.001	0.26 (0.16, 0.44)	<0.001
Δ24Lac Q4	0.18 (0.11, 0.29)	<0.001	0.10 (0.06, 0.17)	<0.001	0.20 (0.11, 0.36)	<0.001
P for trend		<0.001		<0.001		<0.001
**ACLF**						
Δ24Lac per 10%	0.94 (0.92, 0.96)	<0.001	0.92 (0.90, 0.94)	<0.001	0.93 (0.90, 0.96)	<0.001
Categories						
Δ24Lac Q1	Ref	Ref	Ref	Ref	Ref	Ref
Δ24Lac Q2	0.68 (0.46, 1.01)	0.057	0.62 (0.41, 0.95)	0.026	0.52 (0.31, 0.86)	0.012
Δ24Lac Q3	0.30 (0.19, 0.48)	<0.001	0.24 (0.15, 0.39)	<0.001	0.26 (0.15, 0.47)	<0.001
Δ24Lac Q4	0.17 (0.10, 0.29)	<0.001	0.09 (0.05, 0.16)	<0.001	0.17 (0.08, 0.34)	<0.001
P for trend		<0.001		<0.001		<0.001

Model I adjusted for age, sex, LiFe score.
Model II adjusted for age, sex, mean arterial pressure, temperature, 24-hour urine output, albumin, bilirubin, creatinine, INR, vasopressor used, mechanical ventilation, renal replacement therapy.

Supplementary Table 3. Time-dependent competing risk regression analysis in the WMU cohort.

	28-day mortality		90-day mortality	
	**SHR (95% CI)**	**P value**	**SHR (95% CI)**	**P value**
**Cirrhosis**				
ΔLac per 10%	0.91 (0.86, 0.95)	0.001	0.90 (0.87, 0.94)	<0.001
Categories				
ΔLac Q1	Ref	Ref	Ref	Ref
ΔLac Q2	0.82 (0.44, 1.51)	0.520	0.84 (0.48, 1.49)	0.560
ΔLac Q3	0.21 (0.08, 0.53)	0.001	0.26 (0.12, 0.59)	0.001
ΔLac Q4	0.14 (0.05, 0.43)	<0.001	0.17 (0.07, 0.44)	<0.001
**ACLF**				
Δ24Lac per 10%	0.92 (0.87, 0.98)	0.007	0.92 (0.88, 0.95)	<0.001
Categories				
ΔLac Q1	Ref	Ref	Ref	Ref
ΔLac Q2	0.93 (0.48, 1.82)	0.833	0.90 (0.48, 1.68)	0.733
ΔLac Q3	0.29 (0.10, 0.82)	0.020	0.29 (0.11, 0.75)	0.011
ΔLac Q4	0.16 (0.05, 0.50)	0.002	0.16 (0.06, 0.45)	<0.001

CI, confidence interval; Ref, reference; SHR, subhazard ratio
Adjusted for age, sex, mean arterial pressure, temperature, 24-hour urine output, albumin, bilirubin, creatinine, INR, vasopressor used, mechanical ventilation, renal replacement therapy.
Liver transplantation is a competing event.

Supplementary Table 4. Multivariate regression for effect of lactate-clearance (day 3–7) on mortality (derivation cohort).

	In-hospital mortality		28-day mortality		90-day mortality	
	**OR (95% CI)**	**P value**	**HR (95% CI)**	**P value**	**HR (95% CI)**	**P value**
**Cirrhosis**						
ΔLac_{3-7} per 10%	0.84 (0.80, 0.89)	<0.001	0.92 (0.90, 0.94)	<0.001	0.93 (0.92, 0.94)	<0.001
Categories						
ΔLac Q1	Ref	Ref	Ref	Ref	Ref	Ref
ΔLac Q2	0.29 (0.16, 0.51)	<0.001	0.42 (0.29, 0.62)	<0.001	0.43 (0.30, 0.60)	<0.001
ΔLac Q3	0.25 (0.14, 0.44)	<0.001	0.33 (0.22, 0.49)	<0.001	0.35 (0.24, 0.50)	<0.001
ΔLac Q4	0.08 (0.04, 0.16)	<0.001	0.14 (0.08, 0.23)	<0.001	0.19 (0.12, 0.28)	<0.001
**ACLF**						
ΔLac_{3-7} per 10%	0.86 (0.81, 0.91)	<0.001	0.93 (0.91, 0.94)	<0.001	0.94 (0.93, 0.95)	<0.001
Categories						
ΔLac Q1	Ref	Ref	Ref	Ref	Ref	Ref
ΔLac Q2	0.32 (0.16, 0.64)	0.001	0.48 (0.32, 0.72)	<0.001	0.51 (0.35, 0.74)	<0.001
ΔLac Q3	0.31 (0.15, 0.64)	0.001	0.41 (0.27, 0.63)	<0.001	0.43 (0.29, 0.65)	<0.001
ΔLac Q4	0.09 (0.04, 0.20)	<0.001	0.15 (0.09, 0.27)	<0.001	0.20 (0.12, 0.31)	<0.001

Adjusted for age, sex, mean arterial pressure, temperature, 24-hour urine output, albumin, bilirubin, creatinine, INR, vasopressor used, mechanical ventilation, renal replacement therapy.
Q1: <0.03; Q2: ≥0.03 to 0.36; Q3: ≥0.37 to 0.62; Q4: ≥0.63.
